# Validation of VO_2max_ Prediction Using International Formulae for Young Saudi Men

**DOI:** 10.7150/ijms.99236

**Published:** 2024-08-26

**Authors:** Khaled Sayar, Abdullah Bamosa, Lubna Al-Asoom, Ayad Mohammed Salem, Qassim Muaidi

**Affiliations:** 1 Physiology Department, College of Medicine, Imam Abdulrahman Bin Faisal University, Dammam, Saudi Arabia.; 2 Physical Therapy Department, College of Applied Medical Sciences, Imam Abdulrahman Bin Faisal University, Dammam, Saudi Arabia.

**Keywords:** oxygen consumption, physical fitness, exercise test, treadmill test, young adults, reference values

## Abstract

**Objectives:** In this study, we aimed to assess the maximal oxygen uptake (VO_2max_) of young, healthy, non-athletic Saudi men using maximum graded exercise with instant breath-by-breath analysis and to compare this value to the predicted VO_2max_ by international formulae.

**Methods:** In this cross-sectional study, 88 young non-athletic normal-weight Saudi subjects were recruited from Eastern Province of Saudi Arabia with mean age (21.3 ± 1.5 years), weight: (64.7 ± 7.5 kg), height: (172.3 ± 6.1 cm) and body mass index: (21.8 ± 2.1). All subjects were interviewed and examined for eligibility, after which they performed maximum graded exercise testing on a treadmill to obtain VO_2max_. The predicted VO_2max_ was also generated using the following formulae (Edvardsen, Fairbarns, FRIENDS, Hansen, and Jones).

**Results:** The mean measured VO_2max_ was 41.9 ± 7.2 ml/kg/min. While the predicted VO_2max_ using the formulae were: Edvardsen = 66.8 ± 7.9, Fairbarns = 64.1 ± 4.7, FRIENDS = 53.5 ± 2.2, Hansen = 42.8 ± 0.54, and Jones = 50.9 ± 5.1 ml/kg/min. There was a significant difference between all the predicted VO_2max_ and the measured one using the paired t-test (P < 0.001), except for the Hansen's predicted value (P = 0.212). The effect size index (Cohen's d) for the comparison of Hansen's VO_2max_ and measured VO_2max_ was trivial and equal to 0.13. The Bland-Altman test showed good agreement between the measured and Hansen's predicted VO_2max_.

**Conclusion:** This study demonstrated the mean VO_2max_ value of young, healthy, and non-athletic Saudi men. This value was lower than Western values, which might be due to low physical activity or racial differences. Most international formulae overestimate the VO_2max_ in this population, except for the Hansen equation. Therefore, Hansen's predicted VO_2max_ might be the best available reference value for the diagnosis and prognosis of young Saudi individuals undergoing maximum exercise testing.

## Introduction

Physical fitness is an important indicator of individual and population health 1. Evidence showed that a correlation between cardiorespiratory fitness and future cardiovascular and metabolic threats exists early in youth 2. Poor fitness levels among young adults are associated with an increment to 3-6-fold risk in the risk of developing diabetes mellitus, hypertension, and other metabolic syndromes in their middle age 3. Therefore, assessing population fitness levels, particularly youth fitness, is becoming a major concern for governments 4. The best and most objective method for assessing fitness is the evaluation of the level of maximal oxygen consumption, which is designated as (VO_2max_) by cardiopulmonary exercise testing (CPET) 5, 6. However, VO_2max_ can be estimated by indirect methods that rely on data obtained from submaximal exercise, non-exercise (age, weight, height), and hybrid methods 7,8. Most professionals prefer VO_2max_ estimation methods to direct measurements because they are less expensive, less harmful, and more time-effective 9,10. Researchers have invented new methods and equations that rely on novel parameters such as net heart rate (the difference between resting heart rate and exercise heart rate), as demonstrated by Bragada et al. 11, or estimated physical activities, as reported by Sampaio et al. 12. These VO_2max_ prediction methods can provide reference values for the assessment of population fitness and large-scale estimates of health threats 13,14.

Since the 1980s, several equations have been derived for different populations based on data obtained from maximum exercise testing. For example, Jones et al. (1985) developed an equation to predict the VO_2max_ by applying a progressive incremental test to 100 North American individuals with an even age distribution between 15 and 71 years 15. Hansen, et al. (1984) devised Hansen's equation after acquiring data from exercise testing from 265 North American males who were ex-shipyard workers 16. More recently, Edvardson et al., in 2013, published a newly derived equation for predicting VO_2max_. The study population comprised 904 healthy adults of Caucasian race 17. In 2019, Nevil et al. published an improved equation derived from data collected from the Fitness Registry and the Importance of Exercise National Database (FRIENDS) representing 4494 North Americans 18.

However, the existing literature regarding the measurement of VO_2max_ values in Saudi adults is limited, and normal reference values have not yet been established. Most relevant published studies have focused on the cardiorespiratory fitness of Saudi athletes 19, 20 or screening the physical fitness capacity of young or the general male population using questionnaires 21,22. Some have estimated the VO_2max_ of young females based on the maximum exercise and time until exhaustion 23,24. We found only two Saudi cross-sectional studies of the general population that adopted maximum exercise testing and instant gas analysis to assess VO_2max_. One was performed on 137 young schoolboys and showed an absolute VO_2max_ value of 1.2 ± 0.2 L/min and 2.5 ± 0.5 L/min for the age categories 7-9 years and 13-15 years, respectively 25. Similarly, another study performed on young females in Dammam city in 2015 to estimate VO_2max_ of 102 young Saudi females using cycle ergometer and found that the obtained mean VO_2max_ value (27.39 ± 4.06 ml/kg/min) was significantly lower than the international values and the corresponding predicted values by three international equations namely Jones, Wasserman, and Hansen equations 26.

VO_2max_ varies widely depending on age, sex, genetics, ethnicity, lifestyle, exercise training, and health status 27, 28. Ethnicity, as a social construct, encompasses numerous factors such as genetic heritage, cultural practices, and socioeconomic conditions, which can collectively influence an individual's physiological characteristics and overall cardiorespiratory fitness level 29, 30. Studies examining the association between ethnicity and VO_2max_ levels have consistently reported disparities across different racial and ethnic groups. For example, research has shown that non-Hispanic white individuals have higher VO_2max_ levels than non-Hispanic Black individuals and individuals of other ethnic backgrounds 31. The Saudi population has a unique genetic and cultural background. In a local Saudi study, specific single-nucleotide polymorphisms (SNPs) were detected in young Saudi females with low VO_2max_. These SNPs were functionally correlated with the physiological regulation of heart rate, breath tests, cardiac muscle fiber development, and body weight 32. Furthermore, common undiagnosed hemoglobinopathies in young Saudi females that is 3.7 α-globin deletion were also found to be associated with low V̇O_2max_ 33.

Based on the latter discussion of the influence of ethnicity on VO_2max_ and the failure of the international formulae to predict reasonable VO_2max_ values in young Saudi females 26, we hypothesized that the predicted VO_2max_ by the international formulae is different from the actual measured value in young, healthy, normal-weight, non-athletic Saudi males. Therefore, the current project aimed to measure VO_2max_ in young, healthy-weight, non-athletic Saudi males in the Eastern Province of Saudi Arabia by maximum cardiopulmonary exercise testing (CPET) using Bruce protocol on a treadmill and to compare the measured values with the corresponding predicted VO_2max_ values using international prediction equations (Edvardsen, Fairbarns, FRIENDS, Jones, and Hansen) to validate the application of these formulae to Saudi population.

## Methods

This was a cross-sectional study of 88 young, healthy, non-athletic Saudi males recruited from Al-Khobar, Saudi Arabia, between March 2021 and March 2022, using convenience sampling. The study was conducted at the cardiopulmonary exercise testing laboratory of King Fahad Hospital of Imam Abdulrahman Bin Faisal University. Ethical approval was obtained from the Institutional Review Board (IRB) and given the following number (IRB-PGS-2020-01-244, date 21/8/2021).

The sample size was determined using G-Power software (v3.1.9.7) 34, the mean and standard deviation of VO_2max_ of Saudi male adults derived from a previous study 25 and was found to be 85.

The inclusion criteria were being a healthy male, aged 18-24 years, non-athletic, and having a normal body mass index (BMI: 18.50-24.99 kg/m^2^). The exclusion criteria included any subject who failed the Physical Activity Readiness Questionnaire For Everyone (PAR-Q+) 35, failed CPET requirements such as caffeine consumption <12 h, lack of sufficient sleep, or a COVID-19 positive test.

All participants were contacted and explicitly informed of the study procedure. They were interviewed, and the following data were collected: demographic characteristics (age, body weight, height, and BMI), health status, medical and surgical history, drug history, and lifestyle habits. They were also asked to complete the PAR-Q+ test to check their suitability for physical exercise testing.

All eligible participants were scheduled to undergo CPET at the hospital. Before the appointment, the participant signed a written informed consent form and was requested to follow the following instructions: he should not engage in any strenuous physical activity, ensure adequate hydration, and avoid consuming heavy meals and caffeine within 3 h and 12 h, respectively, prior to the testing session. On the day of the procedure, the following assessments were performed: body weight and height recorded on digital weight and portable stadiometer scales (Seca, Hamburg, Germany), BMI, and pre/post-CPET arterial blood pressure (BP) measurements in the sitting position using a manual sphygmomanometer. The exercise testing was conducted by the Quark CPET™ (COSMED® system, Italy), during the period 08:00am-1:00 pm for all subjects. The testing equipment comprised a breath-by-breath gas analyzer, an arterial BP cuff, a pulse oximeter (Pulse Oximetry, COSMED™, Rome, Italy), a treadmill (COSMED, Bitz, Germany), a computer, and software for data analysis. The test was conducted according to the Bruce incremental protocol, which involves running on a treadmill and increasing the velocity and degree every three minutes until exhaustion.

The American College of Sports Medicine (ACSM) guidelines were followed to terminate the study 7. To ascertain the maximal VO_2_ value, a minimum of two out of three criteria must be fulfilled: A plateau in V̇O_2_ despite persistent increment in workload, respiratory exchange ratio of 1:1 or higher, heart rate (HR) within a range of 10 beats of the age-predicted maximum heart rate (HRmax) using the formula [208-0.7X age] 7.

In addition to the direct measurement of VO_2max_, this study employed five established prediction equations to estimate VO_2max_. The equations used in this investigation are Jones Equation 15: VO_2max_ (ml/kg/min) = 0.046 * Height - 0.021 * Age - 0.62 * Sex - 4.31, Hansen Equation 16: VO_2max_ (ml/kg/min)) = 0.0337 * Height - 0.000165 * Age * Height - 1.963 + 0.006 * Weight (Ideal weight) [Ideal weight = 0.79*height-60.7], Edvardsen Equation 17: VO_2max_ (ml/kg/min) = ((4.97 - 0.033 * Age) * 1000) / Weight, FRIENDS Equation 18: VO_2max_ (ml/kg/min) = 45.2 - 0.35 * Age - 10.9 * Sex - 0.15 * Weight + 0.68 * Height - 0.46 * Exercise Mode (for exercise mode: 1 for treadmill and 2 for cycle ergometer), Fairbarns Equation 36: VO_2max_ (ml/kg/min) = 0.023 * Height - 0.031 * Age + 0.0177 * Weight - 0.332. All prediction equations were generated using the data obtained from maximum treadmill exercise testing.

### Statistical analysis

Statistical Package for the Social Sciences version 23 was used for data analysis. Data were normally distributed and are presented as means and standard deviations. A paired t-test was used to compare the values of measured and predicted VO_2max_ using the five equations, and the effect size index (Cohen's d) was calculated. The Bland-Altman test was used to examine the degree of agreement between the measured VO_2max_ and Hansen's predicted value 11,12.

The statistical test was considered significant when α < 0.05.

## Results

**Figure 1:**
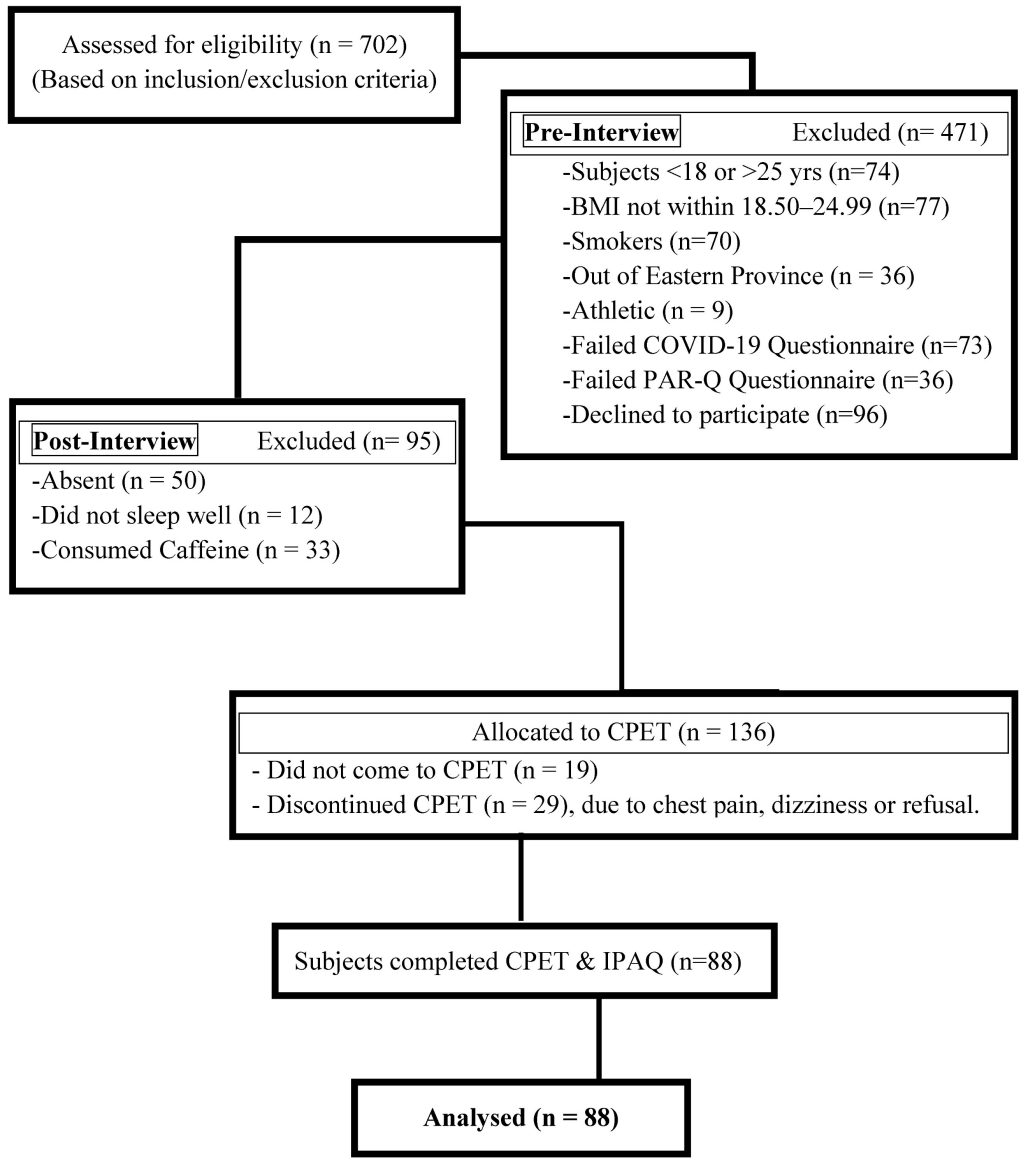
Participant recruitment flowchart.

This study included a total of 88 young, healthy, non-athletic male participants from the Eastern Province of Saudi Arabia for the assessment of VO_2max_, who satisfied the eligibility criteria after screening 702 contacted volunteers, as shown in the recruitment flowchart (Fig. 1).

A total of 449 individuals were excluded based on the predetermined inclusion/exclusion criteria, and the final sample size was 88 participants who were available for analysis. The participants' characteristics including demographic, anthropometric, and exercise data are shown in (Table 1). The overall mean VO_2max_ assessed by using CPET for the entire study population was determined to be 41.9 ± 7.2 ml/kg/min (Table 1). The VO_2max_ of the participants was classified into six categories according to the ACSM, as presented in (Table 2). Among the study sample of 88 participants, a considerable proportion of individuals were classified under the "Very Poor" category, specifically 31.8% (n = 28), and the "Poor" category constituted 22.7% (n = 20) of the participants. Consequently, most participants fell into the "Poor" and "Very Poor" categories, accounting for 54.5% of the study population.

The predicted VO_2max_ (ml/kg/min) using the following equations: Edvardsen, Fairbarns, FRIENDS, Hansen, and Jones were as follows 66.8 ± 7.9 (Edvardsen), 64.1 ± 4.7 (Fairbarns) 53.5 ± 2.2 (FRIENDS), 42.8 ± 0.54 (Hansen), 50.9 ± 5.1 (Jones) ml/kg/min, respectively. The Edvardsen and Fairbarns equations gave the highest values of estimated VO_2max_, whereas the Hansen equation showed the lowest values.

Comparison of the mean measured VO_2max_, and mean predicted VO_2max_ values via international equations using Student's paired t-test showed significant differences in the VO_2max_ obtained by Edwardsen, Fairbarns, FRIENDS, and Jones. These formulae generated significantly higher VO_2max_ values than the actual measured values with P < 0.001. While Hansen's equation showed a predicted VO_2max_ value of 42.8 ± 0.5 ml/kg/min, which was not statistically different from the measured value (P = 0.212). Furthermore, the effect size index Cohen's d for Hansen's predicted VO_2max_ versus measured VO_2max_ was 0.13, which was considered small (Table 3).

**Figure 2:**
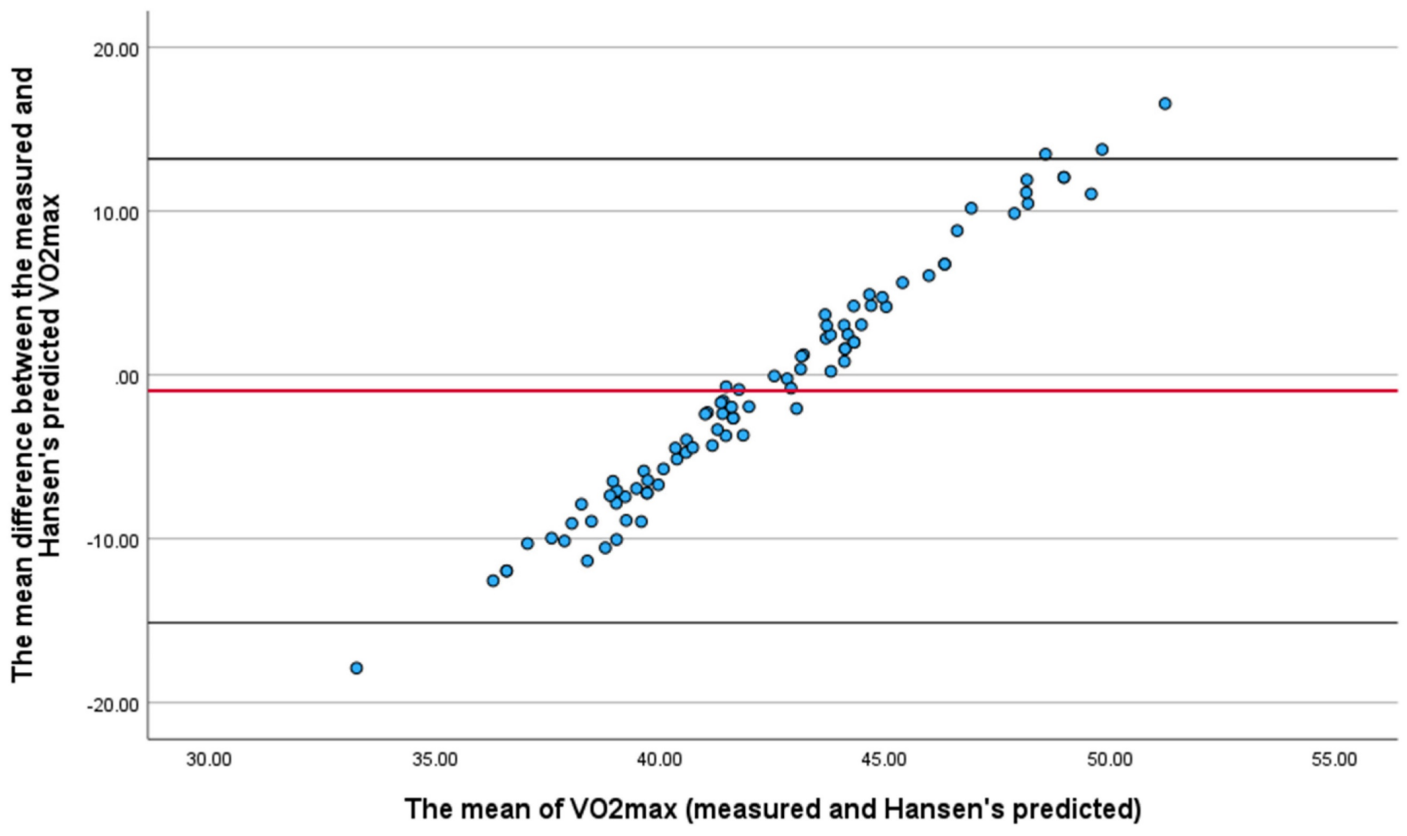
Bland Altman Plot for the measured and Hansen's predicted VO_2max_.

The Bland-Altman plot was applied to study the limit of agreement between the actual VO_2max_ and Hansen's predicted V̇O_2_max (Fig. 2). The mean difference between these two values and the mean of the means of the same values i.e., measured and Hansen's predicted were obtained. Most of the data (approximately 95%) were within the two lines of the limit of agreement, indicating good agreement between the two values.

A one-sample t-test for the difference between the measured and Hansen's predicted VO_2max_ was performed and showed insignificant results (P-value: one-sided = 0.109 and two-sided = 0.212). Therefore, we accepted the null hypothesis that there was no difference between the measured and Hansen's predicted VO_2max_.

Furthermore, to examine the presence of any proportional bias, a linear regression test was performed to determine the difference between the two values (measured and Hansen's predicted VO_2max_) and the mean of the means of these two values. The test was significant (P < 0.001, R = 0.989, adjusted R^2^ = 0.977, and standard error of the estimate = 1.095). Based on the small standard error of the estimate, the percentage of error in Hansen's predicted VO_2max_ in young Saudi men was found to be 2.62%.

## Discussion

Maximal oxygen uptake or VO_2max_, is the best current measure for assessing cardiorespiratory fitness. The assessment of cardiopulmonary fitness is of paramount importance to make individuals aware of their overall fitness status and predict the future risks of cardiovascular diseases 37. Racial differences in the values of VO_2max_ are reported in the literature. Therefore, referrals to international values are not valid, as demonstrated by multiple studies involving different genetic and environmental backgrounds 31. Currently, there are no reference values for the VO_2max_ in Saudi Arabia. Therefore, this study aimed to determine the mean value of VO_2max_ among young, healthy male adults from the Eastern Province of Saudi Arabia using maximum exercise on a treadmill and instant ventilatory assessment and to compare this measured value with the predicted VO_2max_ using international prediction formulae.

The mean value of VO_2max_ of the Saudi young non-athletic males obtained in this study with an incremental Bruce protocol on a treadmill is 41.9 ± 7.2 ml/kg/min. The mean value was found comparable with some internationally reported data such as that reported for Mexican Americans and non-Hispanic whites giving the following values 40.9 ± 0.5, 40.2 ± 0.3, respectively, and higher than the mean value for non-Hispanic blacks which is equal to 37.9 ± 0.6 38. On the other hand, this value was lower than the results reported by studies performed on the Western population. For example, Edvardsen et al. conducted a study on 759 individuals exercised on treadmill to exhaustion and found that the mean V̇O_2_max was 48.6 ± 9.6 ml/kg/min for young healthy males in the age category of 20-29 years 17. Another study by Rossi et al. reported a VO_2max_ mean of 45.0 ± 7.5 ml/kg/min, measured by treadmill on a study group of 18,189 Brazilian participants 39. In China, a study was performed on 964 participants (42% female) with a mean age of 49 ± 12 years to assess V̇O_2peak_ and found that the V̇O_2peak_ of males was 23.75 ± 4.84 ml/kg/min, which is lower than our reported value 40. Although the low Chinese value can be attributed first to the type and modality of the protocol used in their study, which is maximum exercise on a cycle ergometer, and to the recruitment of the older age group, it can also, in part, be explained by the relatively lower body size, weight, and height of the Chinese.

Comparison of our measured VO_2max_ with other local studies, such as Al-Hazzaa et al. and Almakhaita et al., was difficult for multiple reasons, such as different age groups, sex, or exercise modality. Al-Hazzaa et al. reported a VO_2max_ value of 49.6 ± 6.6 ml/kg/min using graded exercise tests on treadmill but for schoolboys with younger age groups (7-15 years) and with no controlled criteria for BMI or physical activity 25. Almakhaita et al. followed similar inclusion and exclusion criteria of our study, but the reported lower V̇O_2max_ value (27.39 ± 4.06 ml/kg/min) was obtained by maximum exercise on a cycle ergometer and for young Saudi females only 26.

Furthermore, according to the VO_2max_ classification of the ACSM, 54.5% (n = 48) of the participants in this study fell into the poor or very poor category. This finding highlights the possible elevated risk of future cardiorespiratory or metabolic diseases in a large segment of the Saudi population. Thorough investigations are needed to determine the main precipitating factors for low fitness, whether it is due to modifiable or unmodifiable factors such as lifestyle, including diet, physical activity, smoking, environmental factors, and genetics, and to design programs to foster cardiorespiratory fitness in the population.

Five internationally recommended equations were used to predict VO_2max_ in the study population. Significantly overestimated VO_2max_ values were found with four of these predictive equations (P < 0.01), namely Edwardsen (66.8 ± 7.9), FRIENDS (53.5 ± 2.2), Jones (50.9 ± 5.1), and Fairbarns (64.1 ± 4.7) ml/kg/min. Contrarily, the Hansen's equation prediction mean (42.8 ± 0.5 ml/kg/min) was found very close to our measured VO_2max_ mean (41.9 ± 7.2 ml/kg/min) and with no significant statistical difference using paired t-test (P > 0.05).

A Korean study reported findings similar to ours as they compared the assessed VO_2max_ of 50 subjects (37 males and 13 females) using a cycle ergometer with predicted using international formulae. They found that all predicted values obtained using Hansen, Jones, and Wesserman overestimated the measured VO_2max_. However, when they implemented a local Chinese equation, it yielded reasonable values that were comparable to the measured values 41. Another study on a large Brazilian cohort of 3119 healthy adult participants, including males and females, found that Jones and Wasserman significantly overestimated the measured VO_2max_. To achieve the closest estimate of VO_2max_, the authors developed their equation, known as the Brazilian equation, which includes age, BMI, and physical activity, and generated values with a high correlation (r = 0.894) with the measured value 42. Almakhaita et al. 2019 also applied Jones, Wasserman, and Hansen equations to predict VO_2max_ for young Saudi females. They compared the predicted values [Jones (35.19 ± 2.12 ml/kg/min), Hansen (33.64 ± 0.24 ml/kg/min), and Wasserman (35.20 ± 0.17 ml/kg/min)] to the actual measured value (27.39 ± 4.06 ml/kg/min). They found a significant overestimation by the three formulae 26.

Based on our current data and reported data from the literature regarding the measured and predicted VO_2max_, we can conclude that implementing international VO_2max_ prediction formulae might be misleading in the diagnosis and prognosis of various clinical conditions in the Saudi population.

However, Hansen's equation predicted reasonable VO_2max_ values for young Saudi men, as represented by good agreement using the Bland-Altman test and the small effect size reflected by Cohen's d value (<0.2). Therefore, Hansen's equation may be used to predict VO_2max_ in young Saudi men. The similarity between Hansen's equation and our measured VO_2max_ might be explained by the fact that the Hansen equation implements a predicted weight rather than an actual weight in the calculation of VO_2max_. Hansen et al. used the formula for the prediction of ideal body weight, which correlates weight to height (ideal weight = 0.79*height-60.7) and found in their experiment that is using the actual weight leads to great variability between the measured and calculated VO_2max_, especially in overweight and obese individuals. In contrast, this variability disappeared when the ideal body weight was implemented in the formula 16. Similarly, our work showed that using ideal body weight instead of actual weight might minimize the variability that exists between different ethnicities. Notably, the recommended utilization of the Hansen equation as a predictor of VO_2max_ in this study was only for young Saudi men and requires further validation in other segments of the Saudi population.

In conclusion, the outcomes of the current study might be the first to report the mean value of VO_2max_ in young non-athletic Saudi males using graded exercise on a treadmill. These values contribute to establishing normal physical fitness values in Saudi Arabia. The current reported value of VO_2max_ was found to be lower than Western values, and it placed more than half of the involved subjects in the categories of poor and very poor. This suggests that either the fitness levels in this specific sector of the Saudi population are low, indicating a considerable risk of cardiovascular and metabolic diseases in the future, or there is a false comparison of VO_2max_ values across different populations. The latter concept may also be supported by overestimating VO_2max_ in young Saudi men using international prediction equations (Edvardsen, FRIENDS, Fairbarn, and Jones). Nevertheless, these findings highlight the need to establish population-based normal data for physical fitness and VO_2max_.

Furthermore, this study found that Hansen equation provides comparable VO_2max_ values to the measured values and can be implemented to predict the fitness of young Saudi men.

## Figures and Tables

**Table 1:** Characteristics of the participants (N=88)

Parameters	Mean ± SD	Min	Max
Age (years)	21.3± 1.5	18	24
Sex	All males		
Nationality	All Saudis		
Weight (kg)	64.7± 7.5	50.0	85
Height (cm)	172.3±6.1	160	186
BMI (Kg/m^2^)	21.8 ±2.1	18.3	24.9
Exercise time until exhaustion (min)	10.6±1.5	7.6	15.1
VO_2max_ (ml/kg/min)	41.9±7.2	24.3	59.5

**Table 2:** Categorical distribution of the study participants according to their measured VO2max following ACSM's classification.

VO_2max_ (ml/kg/min)	N (%)	Mean ± (SD)
Very Poor (≤38.1)	28 (31.8%)	34.1 ± (2.8)
Poor (38.1-42.2)	20 (22.7%)	39.9 ± (1.1)
Fair (42.2-45.7)	18 (20.5%)	44.4 ± (1.1)
Good (45.7-51.1)	11 (12.5%)	48.0 ± (1.6)
Excellent (51.1-56.2)	9 (10.2%)	54.0 ± (1.2)
Superior (≥56.2)	2 (2.3%)	58.1 ± (1.9)
Total	88 (100%)	41.9 ± (7.2)

**Table 3:** Comparison of the directly measured and the predicted VO_2max_ of young, non-athletic Saudi males using five international equations (Edvardsen, Fairbarns, FRIENDS, Hansen, and Jones) using paired Student t-test.

VO_2max_ (ml/kg/min)	Mean ± SD	Mean difference± SD	95% CI of the difference	Two-sided P-value	Cohen's d
Measured	41.9 ±7.2	-	-	-	-
Edvardsen^1^	66.8 ±7.9*	-25.0±10.6	(-27.2) - (-22.7)	P<0.001	2.4
Fairbarns^2^	64.1 ±4.7*	-22.2±8.7	(-24.1) - (-20.4)	P<0.001	2.6
FRIENDS^3^	53.5 ±2.2*	-11.6±7.5	(-13.2) - (-14.5)	P<0.001	1.6
Hansen^4^	42.8±0.5	-1.0±7.2	(-2.5) - (0.6)	P=0.212	0.13
Jones^5^	50.9 ±5.1*	-9.1±9.0	(-11.0) - (-7.1)	P<0.001	1.0

* Values significantly different from the measured VO_2max_ using paired t test FRIENDS: Fitness Registry and the Importance of Exercise National Database. 1: Edvardsen=VO_2max_ (ml/kg/min) = ((4.97 - 0.033 * Age) * 1000) / Weight, 2: Fairbarns= VO_2max_ (ml/kg/min) = 0.023 * Height - 0.031 * Age + 0.0177 * Weight - 0.332. 3: FRIENDS: VO_2max_ (ml/kg/min) = 45.2 - 0.35 * Age - 10.9 * Sex - 0.15 * Weight + 0.68 * Height - 0.46 * Exercise Mode (for exercise mode: 1 for treadmill and 2 for cycle ergometer)), 4: Hansen: VO_2max_ (ml/kg/min) = 0.0337 * Height - 0.000165 * Age * Height - 1.963 + 0.006 * Weight (Ideal weight), [Ideal weight= 0.79*height-60.7], 5: Jones: VO_2max_ (ml/kg/min) = 0.046 * Height - 0.021 * Age - 0.62 * Sex - 4.31.
